# Exosomal lipid composition and the role of ether lipids and phosphoinositides in exosome biology

**DOI:** 10.1194/jlr.R084343

**Published:** 2018-08-03

**Authors:** Tore Skotland, Nina P. Hessvik, Kirsten Sandvig, Alicia Llorente

**Affiliations:** Department of Molecular Cell Biology,* Institute for Cancer Research, Oslo University Hospital-Norwegian Radium Hospital, 0379 Oslo, Norway; Department of Biosciences,† University of Oslo, 0316 Oslo, Norway

**Keywords:** cellular membranes, extracellular vesicles, lipidomics, vesicular transport.

## Abstract

Exosomes are a type of extracellular vesicle released from cells after fusion of multivesicular bodies with the plasma membrane. These vesicles are often enriched in cholesterol, SM, glycosphingolipids, and phosphatidylserine. Lipids not only have a structural role in exosomal membranes but also are essential players in exosome formation and release to the extracellular environment. Our knowledge about the importance of lipids in exosome biology is increasing due to recent technological developments in lipidomics and a stronger focus on the biological functions of these molecules. Here, we review the available information about the lipid composition of exosomes. Special attention is given to ether lipids, a relatively unexplored type of lipids involved in membrane trafficking and abundant in some exosomes. Moreover, we discuss how the lipid composition of exosome preparations may provide useful information about their purity. Finally, we discuss the role of phosphoinositides, membrane phospholipids that help to regulate membrane dynamics, in exosome release and how this process may be linked to secretory autophagy. Knowledge about exosome lipid composition is important to understand the biology of these vesicles and to investigate possible medical applications.

Exosomes and microvesicles are considered to be the main types of extracellular vesicles (EVs) released by living cells (1–5). Exosomes are smaller than 150 nm in diameter and are released from cells after fusion of multivesicular bodies (MVBs) with the plasma membrane; whereas, microvesicles bud from the plasma membrane and are typically 100–1,000 nm in diameter. Intracellular vesicular transport is a main cellular activity that has been thoro­ughly studied for many years (6). In contrast, the vesicular transport of molecules from cell to cell via EVs is a process that has only recently started to be investigated (7–10).

This review will focus on exosomes and, in particular, on their lipids, a topic that has important implications for the biology of exosomes. Exosomes correspond to the intraluminal vesicles (ILVs) of MVBs (also called late endosomes), organelles of the endocytic pathway. These organelles typically have a diameter of 250–1,000 nm and contain numerous ILVs (often ˃30) with a diameter of 50–100 nm (11, 12). The fusion of MVBs with the plasma membrane results in exosome release (**Fig. 1**). MVBs can also fuse with lysosomes and with autophagosomes, the latter resulting in amphisomes (Fig. 1). It should be noted that these organelles can also fuse with the plasma membrane and release their content (13–15). It can be expected that the specific lipid composition of exosomes resembles that of ILVs. Not much is known about the lipid composition of ILVs, but they seem to contain more cholesterol (CHOL), sphingolipids, phosphatidylinositol (PI)-3-phosphate [PI(3)P], and bis(monoacylglycero)phosphate (BMP) (also called lysobisphosphatic acid) than the limiting membrane of MVBs (11, 16). Moreover, the lipid composition of ILVs seems to vary depending on the maturation stage of MVBs, with CHOL being especially enriched in ILVs at early stages and BMP at later stages (16). The high content of CHOL and the low content of BMP in exosomes are in agreement with the idea that exosomes originate from MVBs at earlier stages (16). However, it has also been reported that there are different classes of MVBs, some that fuse with lysosomes and others that give rise to exosomes (17).

**Fig. 1.**
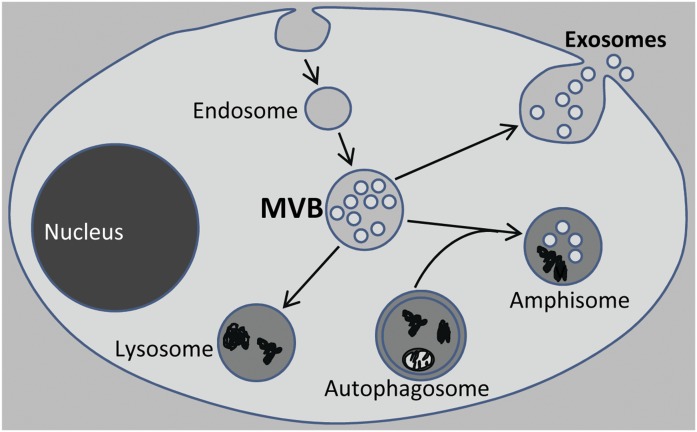
Exosome release. MVBs, also called late endosomes, can fuse with lysosomes, with autophagosomes to form amphistomes, and with the plasma membrane. After fusion of MVBs with the plasma membrane the ILVs of MVBs are released as EVs and then called exosomes.

In this review, we will discuss the available information about the lipid composition of exosomes, including both lipid classes and lipid species. In addition, methodological issues related to the study of exosomal lipids will be discussed. Finally, the role of ether lipids and phosphoinositides (PIPs) in exosome biology will be presented.

## EXPERIMENTAL CONSIDERATIONS IN THE STUDY OF EXOSOMAL LIPIDS

A caveat of exosome research is that the field has developed exponentially during the last decade without having a clear consensus on the optimal methodological approaches. This has caused confusion in the scientific community and, in some cases, results might have been misinterpreted or are based on unsatisfactory purification methods. In this section, these issues are briefly commented on in the context of exosomal lipids, and we refer to our recent review for a more extensive discussion about this topic (18). Based on the studies published so far, exosomes are constituted to a large extent of membrane lipids, although it is possible that minor amounts of other lipids are captured from the cytosol during the formation of ILVs. The exosomal lipid composition should therefore normally be in agreement with the composition of a lipid bilayer. It is well-established that there is an asymmetric distribution of lipid classes in the two leaflets of the plasma membrane. Thus, sphingolipids and phosphatidylcholine (PC) are mostly present in the outer leaflet, and the other lipid classes are mainly located in the inner leaflet (19). One should expect a similar asymmetry in exosomal membranes, at least shortly after exosomes are released from cells. Thus, at least in mammalian cells, quantitative lipid analyses can be used to estimate whether the reported lipid data are in agreement with small vesicles with a bilayer structure. In contrast to exosomes, some EVs in human semen have been reported to contain internal membranes (20), which may complicate such analysis.

Recently, several articles have been published describing errors and limitations in lipidomic analyses and how they can be improved (21, 22). It was stressed that fatty acyl groups with an odd number of carbon atoms are present in limited amounts in cells, and data reporting large amounts of such fatty acyl groups should be interpreted with caution (22).

Several studies have reported high levels of cholesteryl ester (CE), triacylglycerol (TAG), and cardiolipin in exosomal preparations. CE and TAG are normally found in the core of lipid droplets (19) and cardiolipin in the inner membrane of mitochondria (23, 24). Therefore, high levels of these lipids in exosome preparations may indicate that lipid droplets, lipoproteins, or mitochondria have been co-isolated with exosomes. Lipid droplets and mitochondria could potentially be leaked into the extracellular medium and later be co-isolated with exosomes if, during the time exosomes are allowed to accumulate in the medium, some cells die and their membrane is broken. In addition, both organelles can be included in autophagic vesicles (25), secreted by secretory autophagy, and eventually co-isolated with exosomes during the isolation process (26). A combination of lipidomic and proteomic analysis can provide interesting information about the origin of the different types of vesicles that are present in EV preparations. However, there is certainly a need for better methods to purify and characterize the different types of EVs (18, 27–29). When reviewing the literature, it is difficult to evaluate the purity of the exosome preparations and, in this review, some studies where lipid analyses reveal the presence of lipids not expected to be present in exosomes, the plasma membrane, or endosomal membranes are commented on.

It is important to include as much quantitative lipid data as possible in the articles. Lipid amounts are often given as nanomoles per milligram or as mole percent of total lipids and, preferably, both values should be reported. The use of mole percent has, in fact, some advantages, as it eliminates uncertainties in the measurement of protein concentrations that may occur by using, for example, different standard proteins or methods for protein analyses. Furthermore, in order to evaluate the importance of a specific treatment, the amount of the different lipids should be provided, not just the percent changes from cells to exosomes.

Finally, in terms of studies of exosome biogenesis and release, it should be considered that there may be cell type-dependent regulatory mechanisms for the biogenesis and release of exosomes. For example, Trajokovic et al. (30) reported that inhibition or siRNA-mediated depletion of neutral SMase, an enzyme that generates ceramide (Cer) from SM, resulted in reduced secretion of exosomes from Oli-neu cells. Later, a similar effect of neutral SMase was reported in the human embryonic kidney cell line, HEK293 (31), and in T-cells (32). However, the action of neutral SMase does not seem to be required for exosome release in all cell lines tested (33, 34). Furthermore, different MVB subtypes within a single cell line might exploit different pathways, as shown for syntenin-containing exosomes released from MCF-7 cells (35).

## COMPOSITION OF LIPID CLASSES IN EXOSOMES DERIVED FROM KNOWN CELL TYPES

**Fig. 2.**
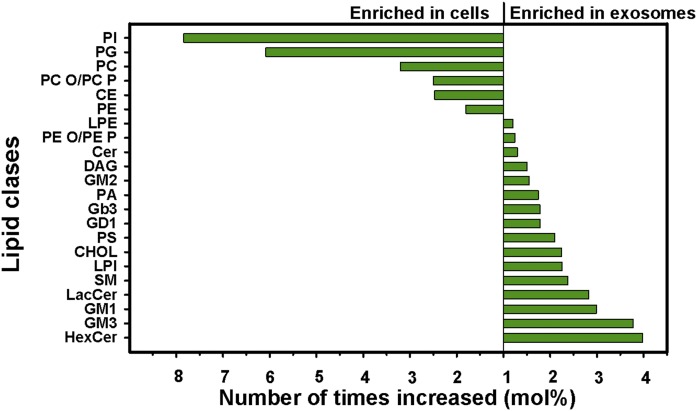
Enrichment of lipid classes in PC-3 cells and PC-3 exosomes. GM1, GM2, GM3, and GD1 are different negatively charged glycosphingolipids. This figure is reprinted from (36) with permission from Elsevier. DAG, diacylglycerol; Gb3, globotriaosylceramide; LacCer, lactosylceramide; LPE, lysophosphatidylethanolamine; LPI, lysophosphatidylinositol; PA, phosphatidic acid; PG, phosphatidylglycerol.

The lipid composition of exosomes described in 10 studies as well as the enrichment factors from cells to exosomes in 8 of them are shown in **Table 1**. Interestingly, these studies show a two to three times enrichment from cells to exosomes of CHOL, SM, glycosphingolipids, and phosphatidylserine (PS). In general, these data show a similar mole percent of phosphatidylethanolamine (PE) in cells and exosomes, and a lower mole percent of PC and PI in exosomes than in their parent cells. Some years ago, we published an extensive quantitative lipid analysis of PC-3 cells and their exosomes (36). The relative changes for 22 lipid classes are shown in (**Fig. 2**), and the quantitative data for 14 of them are shown in the first column of Table 1. Another study of prostate cell lines, including PC-3 cells, also shows a similar enrichment of sphingolipids from cells to exosomes, but the phospholipid classes were grouped together making it difficult to compare these data with other studies (37). Moreover, a lipidomic study of PC-3 cells treated with a precursor (hexadecylglycerol) of ether phospholipids has also been performed. Although this treatment resulted in major changes in the lipidome of cells and their exosomes, similar enrichment factors were obtained from cells to exosomes in cells treated with hexadecylglycerol compared with untreated cells (36, 38), as shown in Table 1 and discussed in (18). Lipidomic analysis of Oli-neu cells (30) and HepG2/C3a cells (39) showed many similarities with PC-3 cells, although Oli-neu cells showed less enrichment of SM and a much higher enrichment of Cer in exosomes (Table 1). Because these studies did not include the CHOL content as percent of total lipids, we have, for comparison, assumed that it constituted 43 mol%, i.e., the same level as in other exosome preparations listed in Table 1.

**TABLE 1.** Lipid composition of exosomes released by individual cell types

Lipids	PC-3 Cells (36)		PC-3 Cells + HG (38)		Oli-neu Cells (30)		HepG2/C3a (39)		B-Lymphocytes (40)		Mast Cells (41)		Dendritic Cells (41)		Reticulocytes (42)		Platelets (45)	Adipocytes (46)
	%	Factor	%	Factor	%*^a^*	Factor	%*^a^*	Factor	%	Factor	%*^b^*	Factor	%*^b^*,*^c^*	Factor	%	Factor	%	%*^a^*
CHOL	43.5	2.3	59	1.7	43	2.3	43	1.9	42.1	3.0	15	1.0	NR	NR	47	1.03	42.5	43
SM	16.3	2.4	9.1	2.0	8.2	1.5	9.7	10.8	23.0	2.3*^d^*	12	2.8	20	2.2	8.4*^d^*	1.31	12.5	12.5
PC	15.3	0.31	10.8	0.33	26.7	0.67	20	0.67	(20.3)*^e^*	(0.76)*^e^*	28	0.66	26	0.6	23.5	1.03	15.9	33
PS	11.7	2.1	6.9	1.2	14.9	3.0	15.6	2.4	(20.3)*^e^*	(0.76)*^e^*	(16)*^e^*	(1.2)*^e^*	(19)*^e^*	(1.6)*^e^*	5.9	0.92	10.5	1.1
PE	5.8	0.55	1.1	0.21	10.9	1.0	7.4	1.2	(14.6)*^e^*	(0.7)*^e^*	24	1.08	26	1.13	12.7	0.84	3.1	4.0
PE ethers	3.3	1.2	4.7	0.81					(14.6)*^e^*	(0.7)*^e^*							3.2	
DAG	1.5	1.5	1.1	0.92														0.8
PC ethers	0.81	0.40	0.7	0.28													1.4	
PG	0.17	0.17	0.1	0.07													NR	
PA	0.16	1.8	0.1	0.33					(20.3)*^e^*	(0.76)*^e^*							NR	
PI	0.13	0.13	0.3	0.16	NR		4.1	0.18	(20.3)*^e^*	(0.76)*^e^*	(16)*^e^*	(1.2)*^e^*	(19)*^e^*	(1.6)*^e^*	2.4	1.1	5.2	2.3
Cer	0.32	1.3	0.7	1.2	NR	3.3	0.63	2.0									0.40	0.2
HexCer	0.76	3.8	2.3	2.1		2.0												0.02
LacCer	0.12	3.0*^f^*	0.7	1.8		NR												
Lipid analysis	MS		MS		MS	NR	MS/GC		TLC		TLC/GLC		TLC/GLC		TLC		MS	MS
Exosome preparations*^g^*	SFM + SUC		SFM + SUC		SFM + SUC + SG		uFCS + SUC + IG		uFCS+ SUC+ SG + immunocapture		uFCS + SUC		uFCS + SUC		uFCS + SUC		SUC + IG	SFM + SUC

%, percent of total lipid quantified; Factor, factor of enrichment from cells to exosomes; DAG, diacylglycerol; NR, not reported; LacCer, lactosylceramide; PA, phosphatidic acid; PG, phosphatidylglycerol; SFM, serum free medium; uFCS, ultracentrifuged FCS; SUC, sequential centrifugation; SG, sucrose gradient; IG, iodixanol gradient.

a Percent CHOL not reported; CHOL set to 43% to better compare the content of other lipid classes with the other data shown.

b Recalculated from their data.

c CHOL not reported; the sum for the other lipid classes is 100% (including LysoPC not included in this table).

d Sum of SM and the glycosphingolipid, GM3.

e Sum for all classes shown in parentheses and having the same numbers.

f Enrichments of other lipid classes are shown in Fig. 2.

g Exosome preparations: methods used to isolate the exosome preparations.

In the studies discussed above, MS was used for lipid quantification. In older studies of exosomes derived from B-lymphocytes (40), mast cells (41), and dendritic cells (41), lipid analyses were performed with TLC and/or gas liquid chromatography (GLC). In these studies, some lipid classes migrated as one band, including PS, later reported to be enriched from cells to exosomes, and PI, for which the opposite has been reported (Table 1). Therefore, it is difficult to compare some of these results with the mentioned MS studies, but, as shown in Table 1, several similarities exist between all these exosome preparations.

The data shown for reticulocytes in Table 1 are from the very first study describing the lipid composition of exosomes and their parent cells (42). Only a slight enrichment of SM and a small decrease in the mole percent of PE are observed when these exosomes are compared with their parent cells. This may be due (at least partially) to the high CHOL content in these cells and/or the nature of the MVBs that fuse with the plasma membrane in reticulocytes, because they have been shown to contain markers of early, not late, endosomes (43). Interestingly, analyses of exosomes isolated after 2, 4, and 7 days of in vitro reticulocyte differentiation revealed major changes in several lipid classes, i.e., PE, PI + PS, and SM (44). In particular, from day 2 to day 7, the PE content increased by approximately 70%, SM decreased by approximately 40%, and the sum of PI and PS decreased by approximately 50%.

The two last columns in Table 1 show the lipid composition of exosomes released from platelets (45) and adipocytes (46). The enrichment factors are not included because the lipid composition of the parent cells was not reported. Compared with other exosomes shown in Table 1, the adipocyte exosomes contain higher amounts of PC and lower amounts of PS (46). In order to make these data fit with a bilayer membrane, almost half of the PC molecules have to be in the inner leaflet. However, the lipid composition of exosomes released from platelets is very similar to exosome preparations from other cells (Table 1). The authors also reported the lipid composition of five other EV fractions with a size somewhat larger than the exosome preparation (the six preparations showed diameters in the range of 106–203 nm, as measured by nanoparticle tracking analyses). This study nicely demonstrates how EVs of different sizes can have different lipid compositions. To illustrate this, the percentage of some lipid classes in exosomes and the percentage range observed in the other five fractions (in parentheses) is shown: CHOL 42.5% (30.8–37.1%), SM 12.5% (6.0–8.0%), PS 10.5% (13.9–18.9%), and PI 5.2% (2.5–3.9%) (45). In agreement with this and with previous results about the rigidity of exosomal membranes (41), analysis of three different types of EVs (exosomes, microvesicles, and apoptotic bodies) from BV-2 cells showed that exosomes have a remarkably higher lipid order than microvesicles or apoptotic bodies (47). In addition, the same group showed that exosomes from several cell lines were less sensitive to detergents than apoptotic bodies and microvesicles (48).

A lipidomic study in LIM1215 colorectal cancer cells identified 500 lipid species (49). These data are not listed in Table 1, as only the relative abundance of lipid species in cells and exosomes were shown. The enrichment factors are very different from those reported in other studies, and the high enrichment of TAG (×24) and CE (×5.7) should have been further investigated to exclude the possibility that lipoparticles and/or lipid droplets were present in the exosomal preparations. A study in U87 glioblastoma cells, Huh7 hepatocellular carcinoma cells, and human bone marrow-derived mesenchymal stem cells of both exosomes and microvesicles has also been published (50). In contrast to many studies, a similar content of SM and PS as percent of total lipids in exosomes and parental cells was reported, whereas the amount of PC and PI were more similar to other studies (50).

To our knowledge, there is only one study where the lipid composition of exosomes secreted from the apical and the basolateral membrane of polarized cells has been compared (51). The exosomes released from the apical and basolateral membrane of polarized murine cortical collecting duct principal cells had mean diameters of 148 nm and 100 nm, respectively. Interestingly, approximately three times more exosomes were obtained from the apical than from the basolateral side, and large differences in the lipid composition of these exosomes were found. However, it is difficult to evaluate these data due to the different relative exosomal lipid composition that is obtained depending on the MS analysis mode (negative or positive ion). Furthermore, the very high content of cardiolipin, a lipid mainly found in the inner membrane of mitochondria, can be an indication of the presence of other types of vesicles or small particles in the exosomal preparations.

As mentioned in the Introduction, BMP can be present in the membranes of the ILVs of MVBs (16). Only a few studies have investigated the presence of this lipid in exosomes. In one study, BMP was reported to be below the detection limit (40), and in the other, this lipid accounted for 0.8 and 1.2 mol% of total phospholipids in exosomes and cells, respectively (41). A third study, based on electron microscopy, found low labeling of BMP in exosomes (16), but it should be noted that this technique is sensitive to fixation and the amount of BMP may have been underestimated. The lack of enrichment of BMP in exosomes is in agreement with the idea that the main destination for BMP is the lysosomes, where it seems to be an important cofactor for sphingolipid catabolism and to contribute to lysosomal stability and integrity (52, 53). Interestingly, Miranda et al. (54) recently reported that BMP-enriched exosomes are released in cells where the endolysosomal membrane is damaged by disruption of the function of the class III PI 3-kinase, Vps34, but the absolute levels of BMP were not provided. It would be interesting to analyze additional exosome preparations with MS methods able to quantify BMP.

Finally, as shown in Table 1, PI is generally found in exosomes. PIPs are phosphorylated derivates of PI that help in specifying organelle identity. In particular, PI(3)P and PI-3,5-bisphosphate [PI(3,5)P_2_] contribute to the identity of early and late endosomes (55, 56). Unfortunately, quantification of PIPs by MS is not straightforward, as PIs phosphorylated at one or two sites have to be separated by chromatography in order to be identified. To our knowledge, there are no studies reporting quantification of PIPs in exosomes so far.

In conclusion, exosomes released from different cell lines have a relatively similar composition of lipid classes in general. Additional experiments in more cellular systems are necessary to further support this idea. It will also be interesting to compare the lipid composition of exosomes and other EVs in other cell types. Moreover, the lipid composition of exosomes, HIV particles (57), and detergent resistant membranes (58) show several similarities, and this is discussed in detail in (18). In agreement with this, a very recent article showed that the lipid composition of encapsulated hepatitis E virus from HEpG2/C3a cells and exosomes released from these cells was very similar (39). Moreover, the lipid content of the hepatitis E-containing particles was very similar to HIV particles (57) and to many of the exosome preparations discussed above.

## COMPOSITION OF LIPID CLASSES IN EXOSOMES DERIVED FROM BIOLOGICAL FLUIDS

Exosomes in biological fluids constitute a heterogeneous population originating from many different cell types. Due to the complex composition of biofluids, the possibility of co-isolating exosomes with other vesicles, particles, or proteins is higher than in more controlled systems, such as cell culture supernatants. In this section, we have used the terms that the authors have chosen to name their EVs.

Only a few studies have investigated the lipid composition of EVs in biofluids. The lipid composition of EVs from seminal fluid, traditionally called prostasomes, was analyzed some years ago by TLC in human (59), horse (60), and boar semen (61). The results of these studies show several similarities with the data discussed in the previous section. However, recent MS analyses of two prostasome fractions of 100 and 50 nm in diameter purified from human seminal fluid (**Table 2**) show that the sphingolipid [SM and hexosylceramide (HexCer)] content varies considerably between the two populations, and that it is higher than that reported for exosomes in Table 1 (62). The third column of Table 2 shows the lipid composition of human urinary exosomes that we reported based on the quantification of 107 lipid species by MS analysis (63). There are some important differences between the lipid composition of urinary exosomes and our data of exosomes derived from PC-3 cells (36). For example, the CHOL content (63 mol%) of urinary exosomes is on the limit of what model membranes have been shown to accommodate (64).

**TABLE 2.** Lipid composition of exosomes from seminal fluid, urine, and nematodes

Lipids	Prostasomes, 100 nm (62)	Prostasomes, 50 nm (62)	Urine (63)	Nematodes (74)
	*%*	*%*	*%*	*^a^*
CHOL	54.8	54.1	63	7
SM	28.6	14.3	11.7	3
PC	2.3	1.3	2.7	4
PS	7.1	5.4	13.2	15
PE	0.6	0.3	<LOQ	13
PE ethers	—	—	4.6	47
PC ethers	—	—	<LOQ	8
PI	1.1	1.3	<LOQ	—
Cer	—	—	0.1	—
HexCer	5.6	23.3	1.9	—
LacCer	—	—	0.8	—
Gb3	—	—	1.1	—
Lipid analysis	MS	MS	MS	MS
Exosome preparations*^b^*	SUC + SEC + SG		SUC	SFM + SUC

%, percent of total lipid quantified; LOQ, limit of quantification; LacCer, lactosylceramide; Gb3, globotriaosylceramide; SEC: size exclusion chromatography; SFM: serum free medium; SG: sucrose gradient; SUC: sequential centrifugation.

a Recalculated from (74); lyso ethers and lyso acyl lipids are not shown.

b Methods used to isolate the exosome preparations.

Blans et al. (65) recently published a study of EVs isolated from human and bovine milk that included information about their phospholipid and TAG composition. The mode diameter of these EVs was reported to be in the range of 147–189 nm, with some vesicles being up to 700 nm. The authors concluded that milk EVs likely contain various EV subsets. Importantly, it was shown that the isolated preparations were not just milk fat globules, as the ratio of TAG to CHOL was lower in the vesicle preparations than in the globules. EVs are also found in blood (66), but we are not aware of any lipidomic study of EVs isolated from whole blood. The complexity of blood and the high content of lipoparticles in this biofluid make it difficult to isolate pure EV preparations, and thus to interpret the lipid composition of blood EVs (67).

## ETHER LIPIDS IN EXOSOMES

Ether lipids have not obtained much attention so far, considering that they constitute a considerable fraction of membrane lipids. Moreover, ether lipids are still hardly mentioned in cell biology text books, even though they have been known for many years. The advances in MS analyses seen in recent years have increased the interest for ether phospholipids. In addition to the discussion below, we refer to the following articles for ether lipid structure and general information about these lipids (68–73).

Interestingly, MS analyses have revealed the presence of ether lipids in exosomes (Tables 1, 2). Ether lipids are mainly found as PC and PE, although these lipids can belong to several phospholipid classes. Alkenyl ethers (vinyl ethers) are often called plasmalogens, and PE plasmalogens can constitute up to 50% of the PE lipids in organs such as brain, kidney, and heart (71). Rather larger amounts of ether lipids have been reported in exosomes released from PC-3 cells (36, 38), mast cells (41), and platelets (45); and surprisingly, all PE species detected in exosomes from human urine were identified as PE ethers (63). Interestingly, it has been shown that the amount of ether lipids in exosomes can be regulated, as shown in a study where an ether lipid precursor was added to PC-3 cells (38).

The lipid composition of exosomes secreted from a nematode parasite has also been analyzed (Table 2) (74). The exosomes released from this parasite showed a remarkable lipid composition with a very high content of PE plasmalogens (47 mol%) and very small amounts of CHOL (7 mol%) and SM (3 mol%) (74). Furthermore, a PE ether with 36 carbon atoms and two double bonds in the hydrophobic chains made up approximately 25% of the total lipids, and these exosomes also contained a remarkably high content of phospholipids with a total of two double bonds in the two fatty acyl chains. The authors speculated that the high plasmalogen content was necessary for the stability of these vesicles and compensated for the low levels of CHOL and SM. The metabolic pathways, including sphingolipid synthesis, are not very well described in nematodes, and it is believed that all sterols are obtained through the diet (74). Interestingly, it has been shown that HIV particles also have a high content of PE ethers (75).

Ether phospholipids have been reported to be involved in membrane trafficking and cell signaling and differentiation, and to possibly function as cellular antioxidants (68, 72). These lipids have also been reported to facilitate membrane fusion (76) and to behave differently from their corresponding fatty acyl analogs. The ether-linked alkyl/alkenyl group probably enters perpendicularly into the membrane; whereas in phospholipids, the two first carbon atoms of the *sn-1* acyl groups are almost parallel to the plasma membrane, while the rest of the acyl chain bends and enters the bilayer. This was first shown for PC alkenyl ethers by magnetic resonance spectroscopy (77). Recently, atomistic molecular dynamics simulations demonstrated a similar behavior of PE alkenyl ethers, resulting in a more densely packed and thicker bilayer (78). The presence of ether lipids in exosomes may therefore have important implications both for their fusion with other membranes and for their stability in the extracellular space. Interestingly, it has been shown that ether glycerophospholipids provided rigidity to the membranes of nematode exosomes, but did not affect their fusion efficiency (74). Concerning the study of ether lipids in PC-3 cells, the cells treated with the ether lipid precursor contained fewer MVBs and fewer ILVs per MVB. It was speculated that this was caused by less mature MVBs, with fewer ILVs, due to an increased fusion of mature MVBs with the plasma membrane (38).

Ether lipids have a unique metabolism; for example, there is an ether phospholipid-specific phospholipase A2 that cleaves off fatty acyl groups from the *sn-2* position (79). This is very relevant for the release of arachidonic acid (C20:4), often present in this position, which is known to be important both in cellular signaling and as a precursor for the synthesis of eicosanoids (80). The level of ether phospholipids has also been shown to be important for the cellular level of CHOL, as it is important for the stability of squalene monooxygenase, a key enzyme in the CHOL synthesis pathway. Surprisingly, it has been shown that both elevated and low amounts of PE plasmalogens lower the levels of CHOL (71).

## COMPOSITION OF LIPID SPECIES IN EXOSOMES

So far, only a few lipidomic studies of exosomes contain quantitative data for large numbers of lipid species, but recent advancements in MS-based lipidomics are expected to facilitate these studies in the future. It has often been reported that the saturation level of the fatty acyl groups in phospholipids is higher in exosomes than in the parent cells. For PC, this is mainly due to an increase in PC 14:0/16:0 and PC 16:0/16:0 (30, 36). In our opinion, it may be even more important to stress the high percentage of phospholipids in exosomes that contain one unsaturated fatty acyl group, e.g., PC 16:0/18:1 and PC 16:0/16:1 are the most abundant PC species in PC-3 cells (36) and Oli-neu cells (30). Moreover, PC 34:1 constituted approximately 60% of the PC species in the two preparations of prostasomes listed in Table 2 (62). In addition, exosomes secreted from PC-3 cells are also enriched in other phospholipids having C18:1 in the *sn-2* position, as shown by the remarkably high content and enrichment of PS 18:0/18:1 (36), for example. This PS species was also the second most abundant species in exosomes isolated from human urine, where CHOL is the most abundant lipid (63). The similar enrichment of CHOL, PS 18:0/18:1, and the very-long-chain sphingolipids in exosomes secreted from PC-3 cells made us hypothesize that there is a hand-shaking (transmembrane coupling between fatty acyl chains present in different membrane leaflets) between these sphingolipids in the outer leaflet and PS 18:0/18:1 in the inner leaflet of the exosomal membranes (36). Further evidence for such a hand-shaking between the two leaflets was obtained by molecular dynamic simulation studies of the interdigitation between these lipids in the presence of CHOL (81). This led us to propose a model for the interaction between these two leaflets that is discussed in detail in a recent review (18).

It should be mentioned that this model assumes that the outer and the inner lipid layers of the exosomal membranes have the same orientation as in the plasma membrane, as can be expected from the mechanism of exosome formation. Therefore, PS, or at least most of it, is assumed to be located in the inner leaflet of exosomes. Several studies have concluded that PS is present in the outer leaflet of exosomes, as it can be detected by using PS-binding proteins, such as annexin 5 and TIM4 (82, 83). PS is, in fact, known to be located in the outer leaflet of activated blood cells, apoptotic bodies, and microparticles or microvesicles released from the plasma membrane, where it acts as an “eat me” signal for macrophages (84). There is increasing evidence that exosomes can transfer signals to other cells and organs, and the presence of PS on the exosome surface is not in agreement with this role, as exosomes would be rapidly removed from circulation. Different isolation protocols may lead to preparations containing different types of EVs, and it should be checked to determine whether the population of EVs showing surface exposure of PS are indeed exosomes. Two recent studies of EVs secreted from human bone marrow mesenchymal stem cells are very interesting regarding this discussion. In one of them, the release of EVs from hypoxic and nonhypoxic cells was investigated. Interestingly, only the vesicles (expected to be a mixture of exosomes and microvesicles) released from hypoxic cells were reported to expose PS on their surface (85). In the other study, three different types of EVs (size diameter of 50–100 nm) were analyzed, and the only vesicle population shown to bind annexin 5 carried low or undetectable levels of exosomal markers, such as CD9, CD81, ALIX, and TSG101 (86). Based on this discussion, we conclude that more studies are needed to establish whether PS is present in the outer leaflet of exosomes when they are secreted, or whether PS translocates during storage.

## PIPs AND EXOSOMES

As mentioned earlier, PIPs are phosphorylated derivates of the membrane phospholipid, PI. Seven PIPs have been identified, and they can be converted into each other by phosphorylation or dephosphorylation of the 3-, 4-, and 5-hydroxyl groups of the inositol head group by phosphoinositide kinases (PIKs) and phosphatases, respectively [for excellent reviews on PIPs, see (55, 56)]. PIPs serve as precursors for specific second messengers involved in signal transduction and, importantly, help to specify organelle identity and regulate membrane dynamics and vesicular transport by recruiting cytosolic proteins containing specific recognition domains to membranes (56, 87). PI(3,5)P_2_, a PIP produced from PI(3)P by the action of the PIP kinase, PIKfyve, is known to be associated with MVBs (88, 89). Due to the role of PIPs in membrane dynamics and vesicular transport, it is not surprising that this lipid may affect exosome secretion. In fact, we have recently shown that inhibition of the formation of PI(3,5)P_2_ by knockdown of PIKfyve with siRNA or by addition of a PIKfyve inhibitor increases exosome secretion in PC-3 cells (26). In addition, this treatment also induced secretory autophagy (26). Autophagy is a degradative pathway that supplies nutrients during starvation and eliminates damaged organelles, aggregated proteins, and invading pathogens (90). This pathway can be induced by various stimuli to maintain cellular homeostasis. Upon induction of autophagy, cytoplasmic cargo is trapped within double-membrane vesicles termed autophagosomes, which then fuse with MVBs to form amphisomes or directly with lysosomes (90) for cargo degradation (Fig. 1). In addition, the autophagic machinery is used to secrete numerous cytoplasmic substrates by a process called secretory autophagy (13, 14). Because the content released by amphisomes might be co-isolated with exosomes during the exosome isolation process, it may be difficult to distinguish exosome secretion from secretory autophagy (26). After PIKfyve inhibition, more MVBs with an increased number of ILVs as well as reduced autophagic degradation were observed in PC-3 cells (26). A possible explanation for this effect could be that the fusion of lysosomes with both MVBs and autophagosomes was impaired under these conditions (Fig. 1). The reported ability of PI(3,5)P_2_ to act as an agonist for the lysosomal Ca^2+^-channel TRPML1 may be related to the impaired fusion of MVBs with lysosomes observed after reduction of the PI(3,5)P_2_ levels (91). In agreement with the role of PIPs in exosome release, it has also been shown that inhibition of the class I PI(3)P kinase or Akt, a downstream component in PI3K signaling, increased the exosomal release of angiopoietin-2 from lung endothelial cells (92).

In addition to the above mentioned results of PIKfyve inhibition, there are other studies that support the existence of a link between autophagy and exosome biogenesis and release (93). Fader et al. (94) showed that induction of autophagy by starvation reduced exosome release. The authors proposed that this was due to an increased fusion of MVBs with autophagosomes, thereby directing MVBs to the degradative pathway. Furthermore, it has recently been reported that depletion of PI(3)P, the major PIP controlling autophagy (55), by inhibition of Vps34 (a class III PI3K), inhibits autophagy initiation, impairs lysosomal degradation, and promotes secretion of exosomes enriched in undigested lysosomal products (54). However, it is not clear whether the undigested lysosomal products were actually inside the exosomes or whether the observed atypical exosome pellet also contained products from secretory autophagy. Moreover, several reports have shown a role for autophagy-related genes (Atgs) in exosome formation and release (95–97). However, the role of some of the investigated Atgs seems to be independent of their function in canonical autophagy. For example, Guo et al. (95) have recently reported that Atg5 depletion reduces exosome release by impairing the disassociation of the V_1_V_0_-ATPase, a proton pump that mediates the acidification of endosomes and lysosomes. Finally, in addition to autophagy studies, there are other reports showing that cellular homeostasis seems to affect the machinery utilized for exosome biogenesis and/or release (98–102).

## CONCLUSIONS

Lipids are important components of exosomes, and several studies demonstrate that they play an important role in the biology of these vesicles. Exosomes are enriched in sphingolipids, CHOL, and PS compared with the donor cells. Interestingly, the lipid composition of exosomes shows similarities with lipid rafts, and exosomes have a higher lipid order and a higher stability against detergents than other EVs. Some exosome preparations have a high content of PE ethers, and more studies are required to investigate the function of these lipids. We also need more quantitative lipidomic studies of exosomes and information about their lipid species. Increasing our knowledge on lipids will help to better understand the biology of exosomes and to facilitate the future exploitation of exosomes in the clinic.
